# Study on Ratio Optimization and Diffusion-Gelation Process of Polymer Grouting Materials for Fracture Filling in Underground Mines

**DOI:** 10.3390/ma17133064

**Published:** 2024-06-21

**Authors:** Xuanning Zhang, Ende Wang

**Affiliations:** Department of Geology, College of Resources and Civil Engineering, Northeastern University, Shenyang 110819, China; 1910354@stu.neu.edu.cn

**Keywords:** grouting, compressive strength, polyurethane material, orthogonal test, gelling, diffusion rule

## Abstract

The existence of fissures poses a serious threat to the safe production of underground mines, and this paper investigates a polymer grouting material for filling fissures in underground mines. To optimise the ratio of polymer grouting materials, this paper designed 16 test groups using the orthogonal test method to find the most reasonable slurry ratio. In order to study the gel diffusion process of polymer slurry in the fissure and to explore the changes of various parameters of the slurry after injection, simulated grouting tests were carried out, and the distribution laws of viscosity, pressure, and diffusion distance of the slurry were discussed. The findings indicate that when the proportion of ethylenediamine polypropylene oxide tetrol: glycerol polyether: catalyst: foam stabiliser is 10:8:0.5:0.4, the polymer grouting material has excellent compressive strength, and the maximum compressive strength can reach 12.31 MPa. Prior to reaching the gel time point, the viscosity of the polymer slurry was nearly constant, which is basically maintained at 0.772 Pa·s under normal temperature and pressure, but after reaching the gel time point, it abruptly rose. As the slurry mass increased, so did the penetration distance and pressure; in the simulated grouting test, when the slurry mass was 400 g, the maximum diffusion distance of the slurry reached 39 cm. Conversely, as the fracture pore size increased, the diffusion distance and pressure of the slurry decreased. Along the diffusion path, the slurry pressure progressively drops, but this change is not synchronised with the diffusion distance’s change. This work can serve as a reference for the configuration of polymer slurry and aid in comprehending the diffusion law of the slurry within the fissure.

## 1. Introduction

Since it is challenging for surface resources to meet society’s needs over a lengthy period of mining in metal mines, underground mining has been on the rise [1,2,3]. The hydrogeological conditions of underground mines have become increasingly complex as mining depth increases. Fissures reduce the strength and stability of the rock body and pose a serious threat to the safety of the lives and property of construction workers by destroying the integrity of the rock body, accelerating its weathering rate, and increasing its water permeability during the mining process [4,5]. Grouting technology plays an indispensable role in seepage control and reinforcement projects, and it is a crucial assurance for the safe production of mine engineering, particularly underground mine engineering, as it is the primary technique of managing underground mine disasters [6,7]. The study of polyurethane grouting material’s configuration and diffusion qualities is especially crucial because it is an organic polymer chemical substance that is being employed more and more in mine fissure filling [8,9,10].

Traditional grouting reinforcement materials like clay, cement, etc. have obvious limitations when compared to chemical grouting materials [11,12]. Since inorganic materials like cement have larger particles, their permeability is limited, they have difficulty penetrating fissures with smaller pore sizes, and their bonding properties are poor. Water can easily wash them away, and it is challenging to accurately control the gel time [13,14,15]. As a result, the range of applications for inorganic grouting materials, like cement, is greatly reduced. Researchers are focusing more and more on chemical grouting materials as efficient water-plugging and strengthening materials. With its ability to swiftly plug holes, stop water leaks, and offer speedy reinforcement, polyurethane is a type of polymer chemical grouting material that is gaining popularity among engineers working on underground mining operations [16,17,18,19]. Common chemical grouting materials include polyurethane as well as water glass, acrylamide, epoxy resin, lignin, and other compounds. Polyurethane grouting materials are among the most often utilised organic grouting materials in projects due to their exceptional all-around performance [20,21,22]. The primary reagents in polyurethane slurry are polyisocyanate and polyols, including polyether polyols and polyester polyols, while the auxiliary reagents are catalyst, surfactant, diluent, retarder, plasticizer, and so on [23,24].

With the advancement of technology in the manufacturing of polymer grouting materials, polyurethane and other polymer-based grouting materials have seen widespread growth and use in recent years, such as in tunnels, highways, railroads, embankments, geological faults, mines, and other projects; polymer grouting materials appear more and more frequently. In order to prevent the occurrence of engineering accidents, as well as to ensure timely treatment of the problems encountered in the project, it is necessary to test grouting materials used on their cementation strength, gelation time, durability, and other aspects of the more stringent requirements. Therefore, an increasing number of researchers are concentrating on the creation and use of grouting materials made of organic polymers, particularly polyurethane materials. A one-step mixing method was used by X. Yu et al. [25] to manufacture a polyurethane water glass composite grouting material. The mechanical and flame-retardant qualities of the composite grouting material are enhanced when sodium silicate is added, and the reaction temperature is also considerably lowered. J. Qian and X. Xiang et al. [26] created a composite grouting material consisting of nano-silica and polyurethane through a two-step polymerization process. The resulting composite grouting material showed outstanding mechanical properties and great resilience to high temperatures when nano-silica was added to the slurry. Q. Zhang et al. [27] looked at how various catalysts affected the microstructure and characteristics of grouting materials made of polyurethane and water glass composite. It was demonstrated that the polyurethane–water–glass composite grouting material exhibited a shorter gel time and a greater compressive strength when the catalyst contents of BDMA and DBTDL were both 0.1%. According to J. Yuan et al. [28], polyurethane grouting has a higher resistance to scouring, which can regulate water flow and avert unexpected water disasters. In a study by S. Samaila et al. [29], it was discovered that the movement of polyurethane grout in sand and gravel was influenced significantly by the size of the pores in the cracks and the grains in the sand and gravel. Polyurethane grout expands into soil by filling existing fissures or forming new ones along the least resistant routes, and it continues to spread into larger cracks in the soil; it plays a positive effect in improving the mechanical properties of weak soil. Y. Wei et al. [30] examined the fatigue behaviour, damage modes, and stress–strain behaviour of polyurethane-cemented body materials under cyclic and static compressive loading. They also developed a physical model to predict the yield stress of rigid polyurethanes with varying densities and proposed a method to determine the size distribution of polyurethane cell-like structures. Despite limited research on the optimal polyurethane slurry ratio, the evolving viscosity behaviour of polyurethane slurry in cracks, and the gel diffusion process, these investigations have significantly improved our comprehension and utilization of polyurethane grouting materials.

The objective of this study is to identify the optimal polyurethane ratio using orthogonal tests, analyse the viscosity variation of the polyurethane slurry with a rotational viscometer, and perform a simulated fissure grouting test to investigate the diffusion process of polyurethane within the fissure. The configuration and gel diffusion properties of polyurethane slurries are the subject of this research, which will serve to direct the design and application of polyurethane slurries in underground mining fissure-filling reinforcement projects.

## 2. Materials and Methods

### 2.1. Grouting Material

This paper utilises a two-component grouting material consisting of component A, which is composed of diphenyl methyl diisocyanate (MDI), and component B, which primarily consists of polyether polyol. The polyurethane slurry is supplemented with auxiliary solvents such as catalysts, foam stabilisers, plasticizers, flame retardants, and diluents. The plasticizers are specifically employed to decrease the hardness of the polyurethane and enhance its thermal properties. Flame retardants are employed to diminish the flammability of polyurethane. Thinners are employed to regulate the viscosity of polyurethane [31]. These additional substances enhance the range of applications for polyurethane. The primary objective of this study is to investigate the durability of polyurethane. Therefore, the catalyst and foam stabiliser are selected as supplementary agents. The precise test materials are displayed in Table 1. An orthogonal test was conducted to ascertain the appropriate proportion of polyether polyol and different additives in component B. In this study, we utilise a combination of ethylenediamine polypropylene oxide tetracosanol (referred to as PPG) and glycerol polyether (referred to as PEG) to create polyether polyol, which is composed of many reagents. The polyurethane slurry was prepared by mixing equal proportions of A and B components using a three-factor, four-level orthogonal test. The specific details of the test may be found in Table 2, while the proportions used are listed in Table 3.

### 2.2. Sample Preparation

#### 2.2.1. Preparation Process of Polyurethane Slurry

Diphenyl methyl diisocyanate (MDI) was poured into a beaker container, and the solution was labelled as component A. The solution was homogeneously stirred at 200 revolutions per minute for 5 min and subsequently kept in a dry-sealed container for more than 20 min. The polyether polyol, catalyst, and foam stabiliser were mixed in the same manner according to the ratios of the orthogonal test protocol, labelled component B. The liquid mixture of component A and component B was poured into a cylindrical PVC tube with a geometric size of 50 × 100 mm^2^, and the whole process was kept at room temperature of 25 °C. The isocyanate was black in colour with a viscosity of 0.15 Pa-s and -NCO mass fraction of 30%, and the polyol was brownish yellow in colour. The isocyanate and polyol were always mixed in equal proportions.

After being left for 1 h, the slurry was allowed to solidify, then the sample was removed from the grinding tool and left to stand for another 24 h to form a standard cylindrical sample. Compressive strength is an important index to measure the performance of grouting materials. A universal testing machine was used to conduct a compression test on polyurethane slurry consolidates marked by varying serial numbers of polyether polyol, catalyst, and surfactant in order to examine how the dosages of these reagents impact the strength of the consolidates. According to the Plastics-Detemination of Compressive Properties [32], 16 groups of samples were tested in compression with 5 samples in each group, totalling 80 samples using the WDW-20 electronic universal testing machine from Shandong Kece Testing Technology Co., Ltd., Jinan, China. The testing machine compressed the samples at a uniform speed of 20 mm/min until the samples were damaged. The expression of compression strength is shown in Equation (1) [33], where *σ* denotes the compression strength in MPa; *F* denotes the instrumental pressure in N, and *A* denotes the original cross-sectional area of the specimen in mm^2^.

(1) $$\sigma =\frac{F}{A}$$

#### 2.2.2. Reaction Principle of Polyurethane Slurry

Isocyanate slurries contain a fairly large number of extremely chemically active unsaturated isocyanate bonds (-NCO), while polymer polyol slurries, which are one of the main agents, contain a large number of hydroxyl groups (-OH). The gel reaction occurs when these two functional groups come into contact. During the gel reaction, the hydroxyl group (-OH) in the polyether polyol reacts with the isocyanate bond (-NCO) in a nucleophilic reaction, resulting in the formation of a urethane group [34]. The carbamate group is a bulky, high-binding energy characteristic group that can be used as a hard chain segment in polymers.

**Figure 1:**
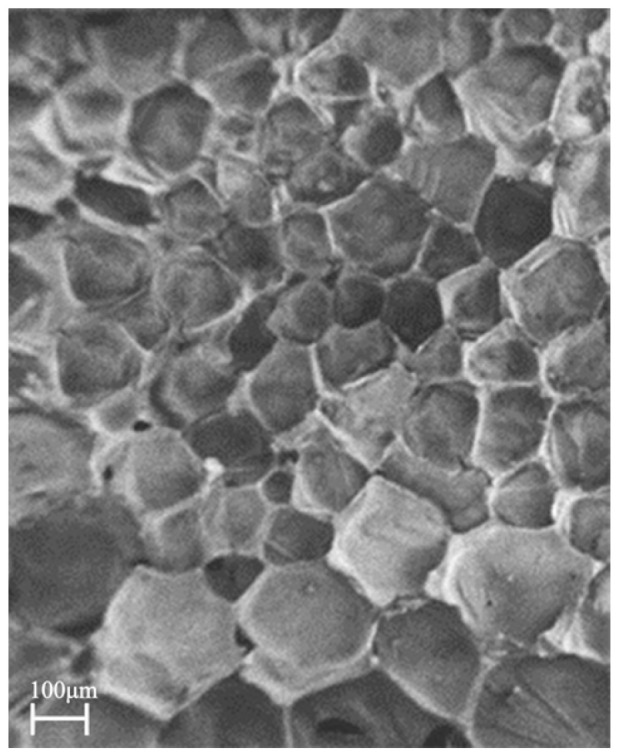
SEM image of the interior of polyurethane material.

In addition to the reaction of the hydroxyl group contained in the polyol, the extremely active and unsaturated -NCO bond contained in the polyisocyanate slurry reacts rapidly with the abundant water in the slurry mixture, which is the foaming mechanism of foamed polyurethane slurry [35]. The carbon dioxide gas released from the foaming reaction is trapped in the reaction mixture, causing it to expand and form a cellular structure, resulting in the formation of polyurethane foam, as shown in Figure 1. The reaction equation for the polyurethane slurry is shown below.

R = N = C = O + R′ − OH → R − NH − COO − R′

Gel reaction

2R − N = C = O + HOH → R − NH − CO − NH − R + CO_2_↑

Foaming reaction

### 2.3. Testing of Polyurethane Slurry

The DNJ-8S Rotational Viscometer from Sanno Instruments in Shenzhen, China was used to measure the viscosity of polyurethane slurry at various time points, while a stopwatch was utilized to record the gel time of the slurry. A motorized rotor in the Rotational Viscometer rotates at a consistent rate to measure the resistance of the liquid being tested, which is then translated into viscosity. To use it, first, pour the right amount of slurry into the viscometer so the rotor is fully submerged; then turn on the rotational viscometer, causing the rotor to spin and create shear stress; finally, once the readings have stabilized, gather and document the viscosity measurements.

At room temperature and pressure, the formula for calculating the viscosity is shown in Equation (2), where *η* denotes the dynamic viscosity, *M* denotes the viscous moment, *ω* denotes the angular velocity of the rotor, *r* denotes the radius of rotation of the rotor, *R* denotes the radius of the outer cylinder, and h denotes the depth of the rotor immersed in the liquid [36].

(2) $$\eta =\frac{M}{4h\omega }\left(\frac{1}{\pi r^{2}}-\frac{1}{\pi R^{2}}\right)$$

**Figure 2:**
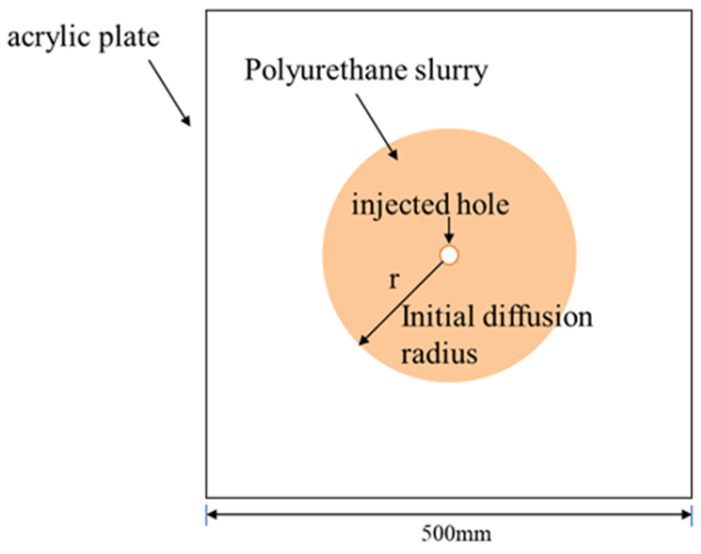
Schematic diagram of polyurethane diffusion in the simulated crack.

The fissure model consisted of two square acrylic plates with a side length of 500 mm, and the spacing between the two plates was adjusted by block rubbers. The slurry was injected through a small hole located at the centre of the plates, and the seepage of the slurry in the simulated fissure was observed, as shown in Figure 2. In addition, the change of pressure was measured by a transducer during the seepage of the polyurethane slurry.

## 3. Results and Discussion

### 3.1. Orthogonal Experimental Analysis

**Figure 3:**
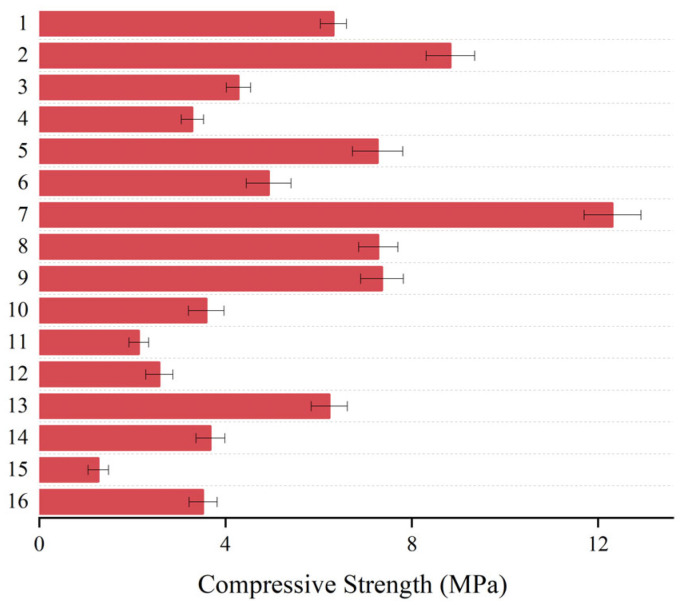
The compressive strength of 16 sets of polyurethane consolidated bodies with different ratios.

Sixteen compression tests were conducted using a three-factor, four-level orthogonal test to determine the uniaxial compressive strength of the consolidates with varying ratios, as illustrated in Figure 3. From this Figure, there is a significant variation in the strength of the cured body for different proportions of polyurethane pastes. The minimum compressive strength value was around 1.27 MPa, and the maximum compressive strength was around 12.31 MPa.

**Figure 4:**
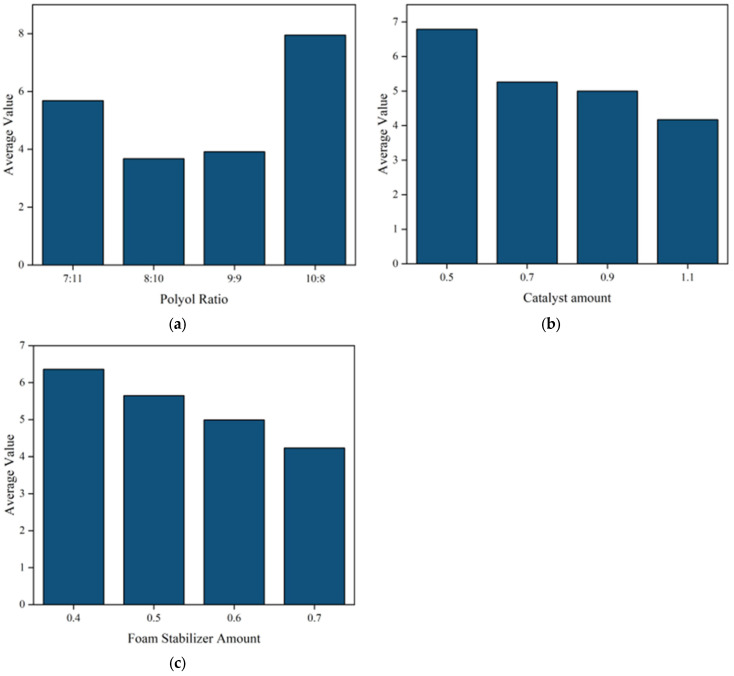
Sensitivity analysis of three factors affecting compressive strength. (**a**) Polyol ratio; (**b**) catalyst amount; (**c**) foam stabilizer amount.

The SPSSAU scientific data analysis platform was used to analyse the range of test data; the mean values of the three influencing factors at different levels are shown in Figure 4, and the R-values and optimal combinations are shown in Table 4. The R-values represent the extreme difference values of the factors, and the extreme difference analyses reflect the sensitivity of the differences between different levels of the factors to the experimental results; a larger extreme difference indicates that the different levels of the factor have a greater impact on the experimental data, and thus the factor can be regarded as the optimal factor.

Figure 4 shows that the polyurethane grouting material has the highest compressive strength at a ratio of 10:8 of PPG to PEG in the polyether polyol, and the compressive strength decreases with the amount of catalyst and surfactant. On one hand, a small amount of catalyst added can balance the foaming and gel reactions during the preparation of polyurethane, resulting in a good internal foam structure. As the amount of catalyst increases, the foaming reaction accelerates, leading to the formation of large collapsing bubbles and more cracks in the cured samples, which has a significant impact on the mechanical properties of the materials. Conversely, using the correct amount of surfactant can enhance the compatibility of the components, prevent foam collapse, and result in a rigid foam with evenly distributed bubble holes and density, ultimately boosting compression strength. Excessive foam stabiliser dosage can lead to a rigid bubble film, resulting in closed-cell foam, decreased compressive strength, and increased brittleness of the material in the system.

Table 4 shows that polyether polyol has the highest discrepancy value of 4.27, significantly surpassing the catalyst and surfactant, indicating its crucial role in regulating the compressive strength of polyurethane materials. The catalyst has the second largest effect on the strength of polyurethane, with an extreme difference value of 2.63, and the surfactant has the smallest effect on the strength of polyurethane, with an extreme difference of 2.13. According to Figure 4 and Table 4, it can be analysed that the compressive strength of the polyurethane slurries can reach the optimum level when the ratio of the polyether polyols (PPG: PEG), catalysts, and surfactants is 10:8:0.5:0.4.

### 3.2. The Gelation Process of Polyurethane Slurry

**Figure 5:**
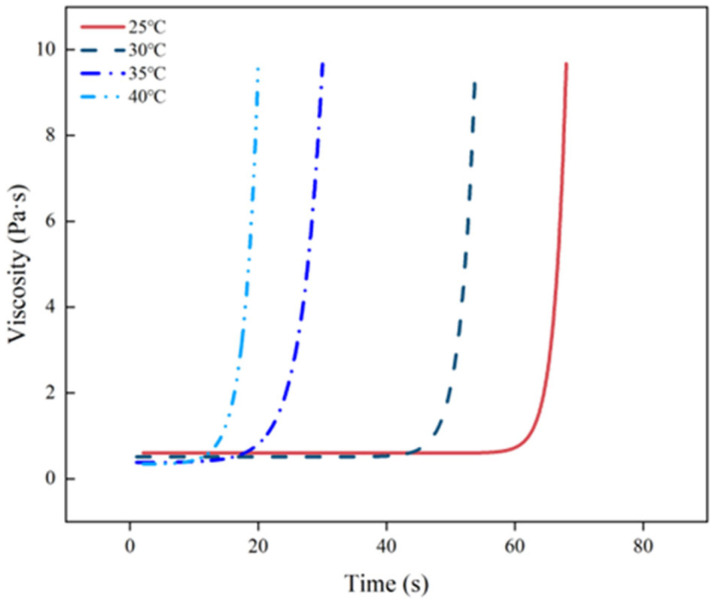
The variation law of viscosity of polyurethane over time.

In Figure 5, the polymer’s viscosity is shown to vary over time at various initial temperatures. The alteration in viscosity can be categorized into two distinct phases. In the first stage, the viscosity of the polymer remains relatively constant until gelation, giving it stable properties. Following the gelling point, the polymer’s viscosity rises, and its fluidity diminishes quickly in the subsequent phase. The point at which the curve changes direction is known as the gelling point.

By analysing data at various temperatures, a model was developed to predict changes in viscosity as time progressed. The findings indicate a rapid growth in polymer viscosity over time, with Equation (3) demonstrating the effectiveness of the fitted curves.

(3) $$\eta =\eta_{0}+A_{0}e^{t/t_{0}}$$

**Figure 6:**
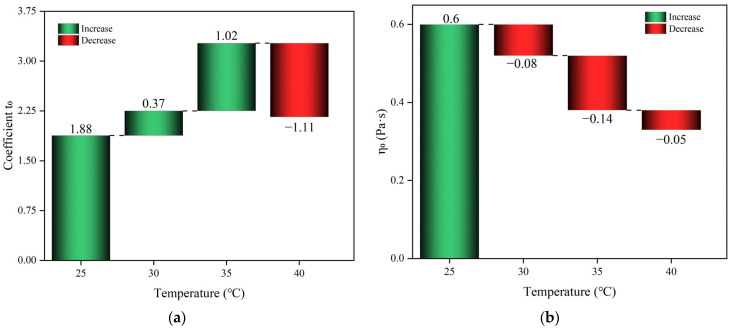
Statistical chart of coefficients for fitting curves. (**a**) The initial viscosity of polyurethane slurry at different temperatures; (**b**) evolution of viscosity change model coefficients.

The viscosity, denoted by *η*, is determined by the coefficients *A*_0_ and *t*_0_, as well as the initial viscosity *η*_0_, over time *t*. Figure 6 and Table 5 display the coefficients of the fitted curves for various starting temperatures. These results reveal that the correlation coefficients between the fitted Equation and the measured data exceed 0.972, suggesting that the model provides a more accurate description of the viscosity evolution of the polyurethane slurry.

**Figure 7:**
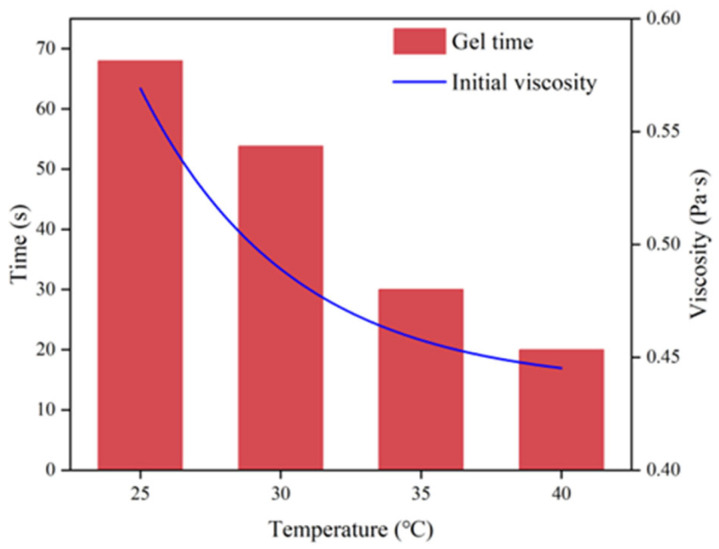
The variation law of gel time and initial viscosity of polyurethane slurry over time.

Figure 7 illustrates a decrease in gel time with increasing initial temperature, accompanied by an exponential decrease in initial viscosity. At a starting temperature of 25 degrees Celsius, the polyurethane slurry has a gel time of approximately 68 s and a viscosity of 0.772 Pascal seconds. Raising the starting temperature to 40 degrees Celsius results in a decrease in gel time to 20 s and viscosity to 0.448 Pa·s for the slurry. As the temperature rises, the polyurethane volume expands, causing molecules to spread out and weakening intermolecular forces, ultimately reducing viscosity. Decreasing temperature leads to a reduction in intermolecular viscous force, resulting in a decrease in viscosity. On the other hand, an increase in temperature increases the activity of the functional groups in the polyurethane molecules, thus accelerating the curing rate of the polyurethane. In contrast, at lower curing temperatures, the polyurethane molecules exhibit decreased activity, resulting in a slower reaction rate and a relatively extended curing time.

### 3.3. Analysis of Slurry Diffusion Process

**Figure 8:**
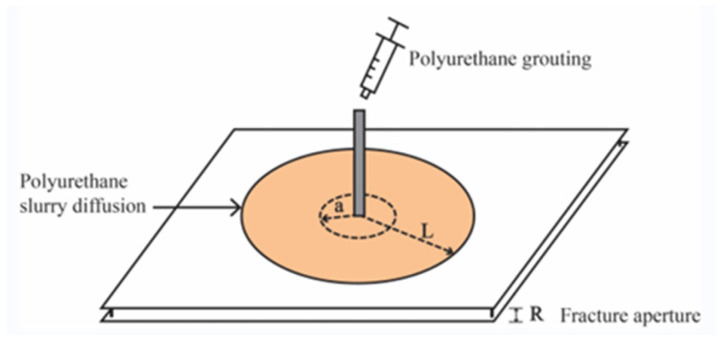
Schematic diagram of slurry diffusion.

Figure 8 shows the diffusion process of polyurethane slurry in planar fissures. It can be found through Figure 8 that the diffusion behaviour of polyurethane slurry in fissures can be divided into three phases: the hydrostatic injection phase, the slurry expansion and diffusion phase, and the curing phase. In the hydrostatic injection stage, the polyurethane slurry is injected into the planar fissure through the grouting pipe, during which the slurry does not expand and the density and viscosity of the slurry are almost unchanged.

As the slurry expands and spreads, the grouting pressure ceases, allowing the polyurethane mixture to continue spreading into the cracks due to the self-expansion force from the chemical reaction, leading to an increase in flow field pressure. As the chemical reaction produces more gas, the amount of gas bubbles in the polyurethane mixture grows, leading to an increase in volume and a decrease in density. The polyurethane grout progresses through the crack wall until it reaches the crucial gelling stage. At this point, the polyurethane slurry changes from a liquid state to a highly viscous semi-solid state, and the slurry stops spreading.

During the curing phase, the polyurethane slurry no longer expands and spreads. The reaction proceeds further by cross-linking. At this point, the durability and chemical resistance of the polyurethane consolidation are enhanced even more.

Therefore, unlike traditional inorganic grouting materials such as cement, the study of the grouting process of polymer materials such as polyurethane cannot be limited to the hydrostatic injection stage but should pay more attention to the expansion and curing stages of the grouting materials. Based on the expansion and curing stages of polymer slurry, the study of the spreading distance of slurry and slurry pressure can be closer to the actual engineering applications.

### 3.4. Diffusion Distance

In the grouting design process, it is crucial to take into account the spread distance of the slurry. In Figure 8, a represents the diffusion distance in the infiltration stage before the polyurethane slurry foams and expands, L represents the diffusion distance after the polyurethane slurry foams and expands, and R represents the pore diameter of the simulated fissure. The spread range of the mixture can be separated into the distance “a” during the infiltration phase and the distance “L” during the expansion phase. Different from the conventional inorganic slurry, the diffusion distance of the polyurethane slurry is mainly determined by the diffusion distance after the expansion stage. Thus, the diffusion distance following the expansion stage of polyurethane is crucial for the practical use of polyurethane slurry, in contrast to the diffusion distance during the penetration stage.

**Figure 9:**
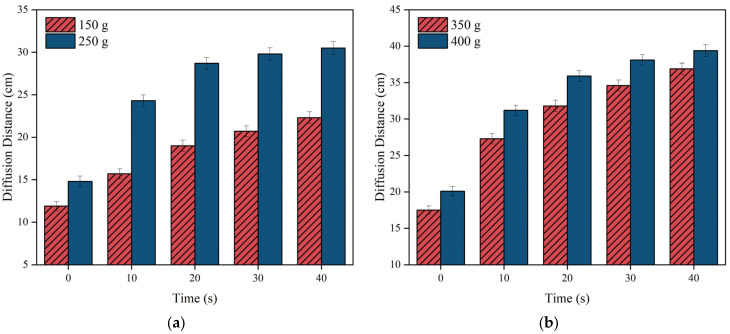
The variation of diffusion distance of slurry over time. (**a**) The grouting volume m is 150 and 250 g, respectively; (**b**) the grouting volume m is 350 and 400 g, respectively.

The average radial distance of polyurethane diffusion was used to track the change in slurry diffusion distance over time at various injection volumes following slurry expansion, as depicted in Figure 9. The graph illustrates a significant increase in the slurry’s diffusion distance within the first 20 s, followed by a gradual decrease until the slurry stops diffusing. In addition, both the slurry diffusion distance and diffusion rate are positively correlated with the grouting volume.

**Figure 10:**
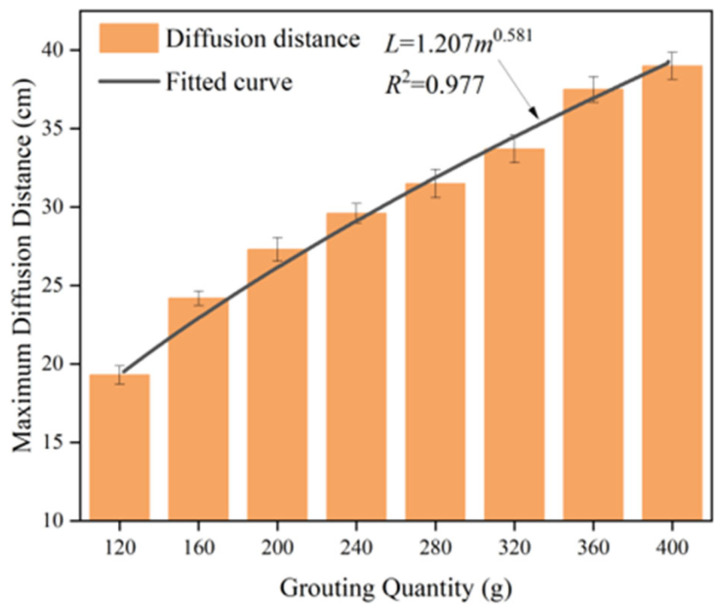
The variation of diffusion distance with the grouting amount.

The relationship between grouting mass and diffusion distance is shown in Figure 10, and it is clear that the diffusion distance increases with increasing grouting mass. With the increase of the quality of the slurry, not only does the volume of the slurry in the crack continue to increase, but also higher pressure is generated inside the slurry, which further promotes the diffusion of the slurry material to the microcrack, thus increasing the diffusion distance. By fitting and analysing the data on the variation of diffusion distance with grouting quality, an evolution model of slurry diffusion distance was obtained. The fitting curve function is shown in Equation (4), where *m* represents the grouting mass in grams and *L* represents the slurry diffusion distance in centimetres. The Coefficient of Determination R^2^ between the fitting formula and the measured data is greater than 0.97, indicating that the model can describe the evolution of polymer diffusion distance well.

(4) $$L=1.207m^{0.581}$$

**Figure 11:**
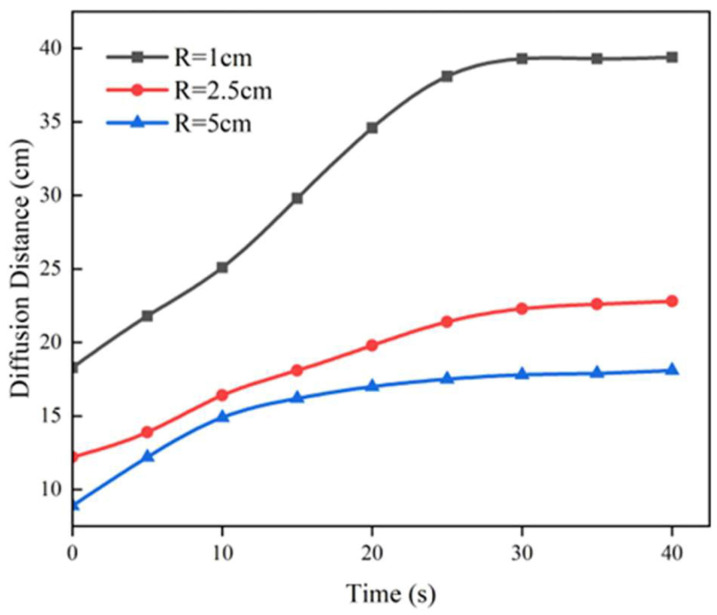
The variation of diffusion distance of slurry in different crack apertures over time.

In Figure 11, the diffusion distance of the polyurethane slurry changes over time in cracks of varying pore sizes, where R indicates the pore size of the cracks being simulated. It is obvious from this figure that the diffusion behaviour of the slurry mainly takes place in the first 25 s, and the diffusion behaviour of the slurry tends to stop after 25 s. The larger the aperture size of the fissure, the smaller the diffusion distance in the initial infiltration stage, and the smaller the final diffusion radius after swelling. From the perspective of slurry expansion, the polyurethane slurry can diffuse more freely during the foaming expansion stage in cracks with larger aperture diameters, and the slurry expansion is subject to fewer constraints, which leads to a smaller slurry diffusion distance.

### 3.5. Slurry Pressure Distribution

**Figure 12:**
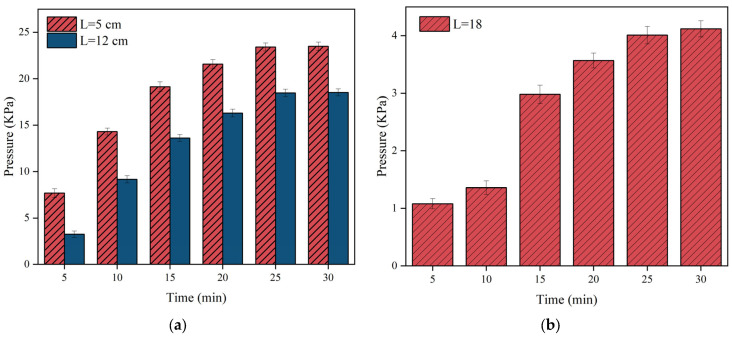
The distribution of slurry pressure when the injection volume m = 150 g. (**a**) The pressure changes over time at monitoring points 5 cm and 12 cm away from the grouting hole; (**b**) the pressure changes over time at monitoring points 18 cm away from the grouting hole.

**Figure 13:**
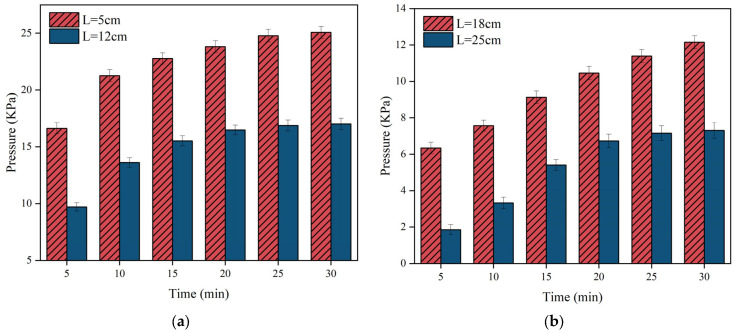
The distribution of slurry pressure when the injection volume m = 250 g. (**a**) The pressure changes over time at monitoring points 5 cm and 12 cm away from the grouting hole; (**b**) the pressure changes over time at monitoring points 18 cm and 25 cm away from the grouting hole.

**Figure 14:**
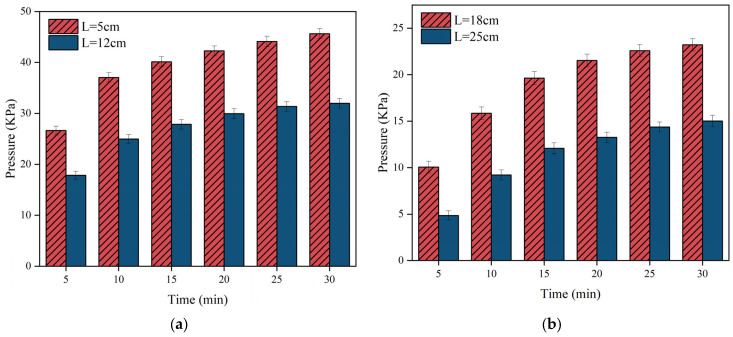
The distribution of slurry pressure when the injection volume m = 350 g. (**a**) The pressure changes over time at monitoring points 5 cm and 12 cm away from the grouting hole; (**b**) the pressure changes over time at monitoring points 18 cm and 25 cm away from the grouting hole.

Figure 12, Figure 13 and Figure 14 illustrate the variation of pressure field time for different grouting volumes. It is clear that the pressure increases gradually with time. However, the change of pressure lags behind the change of slurry diffusion distance. The spreading distance of the slurry stabilises after 30 s and it no longer spreads, but the pressure increase continues for up to 30 min. During the pressure increase, the polyurethane slurry starts to solidify gradually, leading to an increase in strength and chemical stability.

Figure 12 illustrates that the pressure rises predominantly within the initial 15 min, then gradually levels off after 20 min. In Figure 12b, when the grouting volume is 150 g, the spreading distance of the slurry does not reach 25 cm, so the pressure is always zero at the position of *L* = 25 cm. Figure 13 illustrates that as the distance from the grouting hole decreases, the pressure increases, with a maximum pressure reaching nearly 25 KPa. By comparing Figure 12, Figure 13 and Figure 14, it is found that compared to the magnitude of the pressure when the grouting volume is at 150 g and 250 g, the overall distribution of the pressure is the largest when the grouting volume is 350 g, with a maximum pressure of up to 45 KPa at the location near the grouting hole (*L* = 5 cm).

The reason for the different behaviours between Figure 12, Figure 13 and Figure 14 is mainly due to the difference in slurry quality. As the slurry mass increases, the overall pressure increases. The reason for this phenomenon is that in the limited and closed or semi-closed space of the fracture, the available space for the slurry decreases as it fills as more polyurethane slurry is injected into the fracture. At the same time, the injected polyurethane slurry begins to undergo a chemical reaction that results in swelling, further occupying the remaining space within the crack. This expansion, coupled with the continuous injection of the slurry, has a multiplier effect on the increase in pressure.

**Figure 15:**
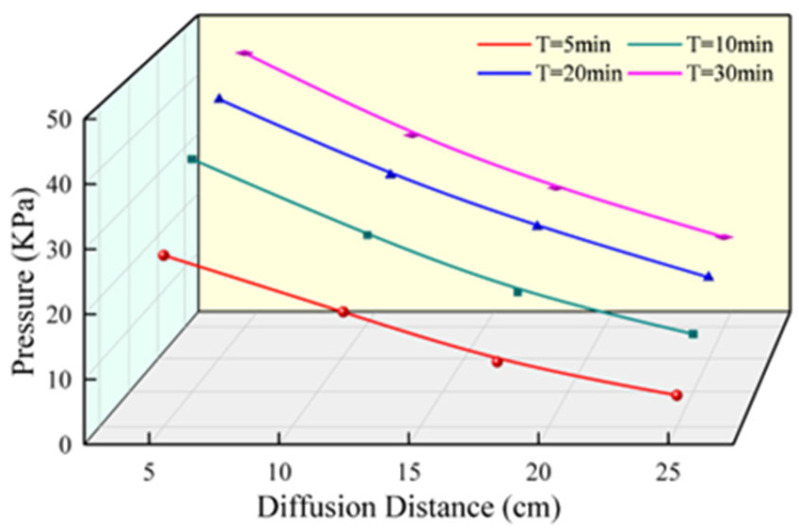
The variation of pressure with diffusion distance.

**Figure 16:**
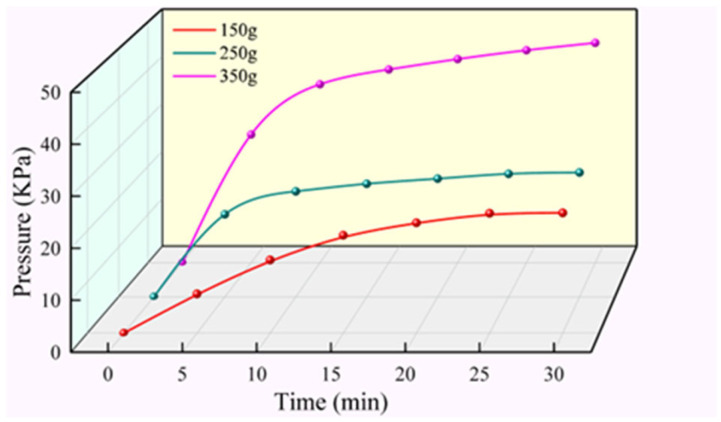
The variation law of pressure with time near the grouting hole (*L* = 5 cm).

By comparing Figure 11 with Figure 14, Figure 15 and Figure 16, it is found that the change of slurry pressure is not synchronous with the development of diffusion distance, and the slurry pressure is still increasing in the case that the slurry stops diffusing. When the polyurethane slurry was injected into the fissure with a pore diameter of 5 centimetres (R = 5 cm), the slurry gradually spread until the spreading distance of the slurry almost stopped increasing after 10 min of slurry injection, as shown in Figure 11. In Figure 14, Figure 15 and Figure 16, it is found that the pressure still increases after the time reaches 10 min. The penetration distance of the slurry is directly impacted by the gel time, while the slurry pressure change is independent of the gel time, explaining this occurrence.

The variation of pressure with diffusion distance is shown in Figure 15. The relationship between pressure and diffusion distance is evident, as the pressure decreases gradually with the increase in slurry diffusion distance. When the polyurethane slurry moves in the fissure, the friction between the slurry and the inner wall of the fissure generates resistance, resulting in pressure loss, and the larger the diffusion distance is, the more resistance generated by friction accumulates, resulting in greater pressure loss.

Figure 16 demonstrates the variation of pressure near the grouting holes with time for different grouting volumes. As can be seen from the Figure, the rate of pressure growth is not the same for different grouting volumes, and the rate of pressure growth increases as the grouting volume increases. When the grouting volume was 350 g, the pressure growth rate was relatively maximum, and within the first 10 min of the spreading of the slurry, the pressure growth rate was about 3.7 KPa/min; at 10 to 15 min, the pressure growth rate was about 0.62 KPa/s, and after 15 min, the pressure was almost no longer increased. In addition, the maximum pressure increased along with the increase of grouting volume, and when the grouting volume rose from 150 g to 350 g, the maximum pressure also rose from 23.51 KPa to 45.65 KPa.

## 4. Discussion

In the process of underground mining, the presence of fissures reduces the strength and stability of the rock body, which in turn poses a serious threat to the safety of engineers. To solve the problem of fissures in underground mines, a high-molecular polymer grouting material was prepared in this study. A set of compositional schemes of polymers with excellent strength properties was determined by orthogonal tests. In order to further understand the gel diffusion law of the high-molecular polymer material, this study carried out simulated fissure filling tests on the polymer material and conducted a series of studies on the viscosity, pressure distribution, diffusion distance, and other parameters of the slurry, which led to the following conclusions:

- The orthogonal test results indicate that a ratio of 10:8:0.5:0.4 for ethylenediamine, polypropylene oxide tetrol, glycerol, and polyether catalyst foam stabilizer produces polyurethane grouting material with optimal compressive strength in this experiment. The polyether polyol had the greatest effect on the compressive strength of the polyurethane, with a maximum strength value of 12.31 MPa.
- The polyurethane slurry’s initial viscosity and solidification time were notably reduced as the starting temperature rose, resulting in a gel time ranging from 20 to 68 s; the change rule of viscosity with time was not linear. The consistency of the polyurethane mixture remained relatively stable until it reached the gelation point; however, when the gelation point was reached, there was a significant increase in viscosity and the mixture quickly solidified.
- The movement process of polyurethane slurry in the fissure is divided into the infiltration stage, expansion stage, and curing stage. As the grouting mass increases, the slurry spreading distance also increases exponentially, while the spreading distance decreases as the pore diameter of the fissure increases.
- The slurry pressure decreases with increasing seepage distance. The change in slurry pressure is not synchronised with the change in diffusion distance, and the change in slurry pressure still occurs when the slurry stops diffusing. This is because when the polymer slurry stops spreading, the foaming reaction within the slurry is not finished, and the large amount of gas produced by the foaming reaction cannot easily escape in a closed or semi-closed fissure environment, resulting in a continuous change in slurry pressure.

Compared with cement and other inorganic grouting materials, high-molecular polymer materials have the advantages of short and controllable gel time and stronger permeability, so they have unique advantages in the filling of small cracks and the sealing of underground water influx. Future research on high-molecular polymer materials will be more inclined to improve the performance of the material and the preparation process to meet the increasingly stringent environmental requirements and higher performance needs.

## Figures and Tables

**Table 1:** Type of test material.

Grouting Material	Type	Dosage (g)	Specification
Isocyanate	MDI	18	Analytical reagent
Polyether polyol	GE-220	18	Analytical reagent
Catalyst	DBTDL	0.5–1.1	Analytical reagent
Foam stabilizer	PMX-200	0.4–0.7	Analytical reagent

**Table 2:** Three-factor and four-level orthogonal test scheme.

Factor Level	Polyether Polyol (PPG:PEG)	Catalyst	Foam Stabilizer
1	7:11	0.5	0.7
2	10:8	0.7	0.6
3	9:9	0.9	0.5
4	8:10	1.1	0.4

Note: The ratio between PPG and PEG is a factor that affects the performance of polyurethane materials.

**Table 3:** Polyurethane slurry ratio scheme.

Number	PPG:PEG	Catalyst	Foam Stabilizer
1	7:11	0.5	0.7
2	7:11	0.7	0.6
3	7:11	0.9	0.5
4	7:11	1.1	0.4
5	10:8	0.5	0.6
6	10:8	0.7	0.7
7	10:8	0.9	0.4
8	10:8	1.1	0.5
9	9:9	0.5	0.5
10	9:9	0.7	0.4
11	9:9	0.9	0.7
12	9:9	1.1	0.6
13	8:10	0.5	0.4
14	8:10	0.7	0.5
15	8:10	0.9	0.6
16	8:10	1.1	0.7

**Table 4:** Range and optimal combination.

Factor	Optimum Proportion	R
PPG:PEG	10:8	4.27
Catalyst	0.5	2.63
Foam stabilizer	0.4	2.13

Note: R is the range value, representing the maximum difference in experimental results produced by the same factor at different levels. The larger the R, the deeper the impact of this factor on the experimental results.

**Table 5:** Statistical table of coefficient and correlation of viscosity change model.

Temperature/°C	A_0_	Correlation R^2^
25	1.415 × 10^−15^	0.972
30	2.912 × 10^−10^	0.983
35	9.527 × 10^−4^	0.986
40	8.936 × 10^−4^	0.975

## Data Availability

The data presented in this study are available in the article.

## References

[1] Carvalho F.P. (2017). Mining Industry and Sustainable Development: Time for Change. Food Energy Secur..

[2] Izatt R.M., Izatt S.R., Bruening R.L., Izatt N.E., Moyer B.A. (2014). Challenges to Achievement of Metal Sustainability in Our High-Tech Society. Chem. Soc. Rev..

[3] Dong L., Tong X., Li X., Zhou J., Wang S., Liu B. (2019). Some Developments and New Insights of Environmental Problems and Deep Mining Strategy for Cleaner Production in Mines. J. Clean. Prod..

[4] Yang Z., Zhao Q., Liu X., Yin Z., Zhao Y., Li X. (2022). Experimental Study on the Movement and Failure Characteristics of Karst Mountain with Deep and Large Fissures Induced by Coal Seam Mining. Rock. Mech. Rock. Eng..

[5] Nian G., Chen Z., Zhu T., Zhang L., Zhou Z. (2024). Experimental Study on the Failure of Fractured Rock Slopes with Anti-Dip and Strong Weathering Characteristics under Rainfall Conditions. Landslides.

[6] Bai E., Guo W., Tan Y. (2019). Negative Externalities of High-Intensity Mining and Disaster Prevention Technology in China. Bull. Eng. Geol. Environ..

[7] Bo L., Yang S., Liu Y., Zhang Z., Wang Y., Wang Y. (2023). Coal Mine Solid Waste Backfill Process in China: Current Status and Challenges. Sustainability.

[8] Li S., Liu R., Zhang Q., Zhang X. (2016). Protection against Water or Mud Inrush in Tunnels by Grouting: A Review. J. Rock Mech. Geotech. Eng..

[9] Wu L., Wu Z., Weng L., Liu Y., Liu Q. (2023). Investigation on Basic Properties and Microscopic Mechanisms of Polyacrylate Latex Modified Cement Grouting Material for Water Blocking and Reinforcement. Constr. Build. Mater..

[10] Wang S., Gong R., Li Z., Yuan C., Jiang G., Wang J., Chen L., Ye C. (2019). Water-Blocking Nano-Composite Cement-Based Grouting Materials. Appl. Nanosci..

[11] Cheng M., Zeng Y., Chen L., Yang H. (2022). Long-Term Compressive Strength and Hydraulic Property of Nanosilica-Improved Sand in Different Soaking Environments. Mater. Today Commun..

[12] Ramkumar V.R., Murali G., Asrani N.P., Karthikeyan K. (2019). Development of a Novel Low Carbon Cementitious Two Stage Layered Fibrous Concrete with Superior Impact Strength. J. Build. Eng..

[13] Yao H., Xie Z., Huang C., Yuan Q., Yu Z. (2021). Recent Progress of Hydrophobic Cement-Based Materials: Preparation, Characterization and Properties. Constr. Build. Mater..

[14] Zhou C., Ren F., Wang Z., Chen W., Wang W. (2017). Why Permeability to Water Is Anomalously Lower than That to Many Other Fluids for Cement-Based Material?. Cem. Concr. Res..

[15] Jing M., Ni G., Zhu C., Li Z., Wang G., Wang Z., Huang Q. (2023). Effect of New Modified Materials on the Microscopic Pore Structure and Hydration Characteristics of Sealing Materials in Coal Seam Boreholes. Constr. Build. Mater..

[16] Sabri M.M.S., Vatin N.I., Alsaffar K.A.M. (2021). Soil Injection Technology Using an Expandable Polyurethane Resin: A Review. Polymers.

[17] Cui Y., Tan Z., Han D., Song J. (2022). Investigation and Application of a High Performance Grouting Material in Water-Rich Silty Fine Sand Stratum. Constr. Build. Mater..

[18] Xue Y., Kong F., Li S., Qiu D., Su M., Li Z., Zhou B. (2021). Water and Mud Inrush Hazard in Underground Engineering: Genesis, Evolution and Prevention. Tunn. Undergr. Space Technol..

[19] Qin Z., Shi Q., Qin D., Wang H., Luo Y., Wang W. (2023). Performance Comparison of Geopolymer and Clay-Cement Grouting Pastes and Goaf Effect Evaluation of Grouting Backfilling Method. Front. Mater..

[20] Yang Z., Zhang X., Liu X., Guan X., Zhang C., Niu Y. (2017). Flexible and Stretchable Polyurethane/Waterglass Grouting Material. Constr. Build. Mater..

[21] Jędrzejczak P., Collins M.N., Jesionowski T., Klapiszewski Ł. (2021). The Role of Lignin and Lignin-Based Materials in Sustainable Construction—A Comprehensive Review. Int. J. Biol. Macromol..

[22] Wang C., Guo C., Du X., Shi M., Liu Q., Xia Y. (2021). Reinforcement of Silty Soil with Permeable Polyurethane by Penetration Injection. Constr. Build. Mater..

[23] Zia K.M., Bhatti H.N., Ahmad Bhatti I. (2007). Methods for Polyurethane and Polyurethane Composites, Recycling and Recovery: A Review. React. Funct. Polym..

[24] Wegener G., Brandt M., Duda L., Hofmann J., Klesczewski B., Koch D., Kumpf R.-J., Orzesek H., Pirkl H.-G., Six C. (2001). Trends in Industrial Catalysis in the Polyurethane Industry. Appl. Catal. A Gen..

[25] Yu X., Liu L., Wang Y., Bai G., Zhang Y. (2021). Effects of Foaming and Drainage Behavior on Structure and Properties of Polyurethane/Water Glass (PU/WG) Grouting Materials for Coal Mines. Adv. Civil Eng..

[26] Xiang X.J., Qian J.W., Yang W.Y., Fang M.H., Qian X.Q. (2006). Synthesis and Properties of Nanosilica-reinforced Polyurethane for Grouting. J. Appl. Polym. Sci..

[27] Zhang Q., Hu X., Wu M., Zhao Y., Yu C. (2018). Effects of Different Catalysts on the Structure and Properties of Polyurethane/Water Glass Grouting Materials. J. Appl. Polym. Sci..

[28] Yuan J., Chen W., Tan X., Yang D., Zhang Q. (2020). New Method to Evaluate Antiwashout Performance of Grout for Preventing Water-Inrush Disasters. Int. J. Geomech..

[29] Saleh S., Yunus N.Z.M., Ahmad K., Ali N. (2019). Improving the Strength of Weak Soil Using Polyurethane Grouts: A Review. Constr. Build. Mater..

[30] Wei Y., Wang F., Gao X., Zhong Y. (2017). Microstructure and Fatigue Performance of Polyurethane Grout Materials under Compression. J. Mater. Civ. Eng..

[31] Naudts A. (2003). Irreversible Changes in the Grouting Industry Caused by Polyurethane Grouting: An Overview of 30 Years of Polyurethane Grouting. Proceedings of the Grouting and Ground Treatment.

[32] Aktaş M., Karakuzu R. (2009). Determination of Mechanical Properties of Glass-epoxy Composites in High Temperatures. Polym. Compos..

[33] Xu S., Wang S., Zhong Y., Zhang B., Zhang J., Wang Y., Zhao L. (2020). Compression Characteristics and Constitutive Model of Low-Exotherm Modified Polyurethane Grouting Materials. Adv. Civ. Eng..

[34] Yeh J.-M., Yao C.-T., Hsieh C.-F., Yang H.-C., Wu C.-P. (2008). Preparation and Properties of Amino-Terminated Anionic Waterborne-Polyurethane–Silica Hybrid Materials through a Sol–Gel Process in the Absence of an External Catalyst. Eur. Polym. J..

[35] Rahman M.M., Hasneen A., Lee W.-K., Lim K.T. (2013). Preparation and Properties of Sol–Gel Waterborne Polyurethane Adhesive. J. Sol-Gel Sci. Technol..

[36] Liu X., Wang J., Huang K., Li F. (2019). Experimental Study on Dynamic Water Grouting of Modified Water-Soluble Polyurethane. KSCE J. Civ. Eng..

